# A Prospective Study of Patients Presenting With Pre-sternal Keloids

**DOI:** 10.7759/cureus.61695

**Published:** 2024-06-04

**Authors:** Ved Prakash Rao Cheruvu, Manal M Khan, Gaurav Chaturvedi, Deepak Krishna, Rahul Dubepuria, Abhinav Singh, Anusha Devalla

**Affiliations:** 1 Department of Burns and Plastic Surgery, All India Institute of Medical Sciences, Bhopal, IND; 2 Department of Obstetrics and Gynecology, All India Institute of Medical Sciences, Hyderabad, IND

**Keywords:** intralesional injections, triamcinolone acetonide, pre-sternal keloids, pathological scarring, keloids

## Abstract

Introduction: Keloid represents a pathological form of scarring. They are very common in the anterior chest area; nearly 50% of all keloids occur in this location. One of the reasons for this is that folliculitis and acne, known for triggering the development of keloids, are common on the anterior chest. The other reason is the tension load in this area due to the frequent movements of the upper limbs and the respiratory movements. These movements stretch the skin of the anterior chest horizontally. When this cyclical tension is imposed on the anterior chest wounds, there is an exacerbation and prolongation of the inflammation in the reticular dermis of the wound. These stresses induce the growth of keloids along the prevailing lines of skin tension.

Materials and methods: We performed a prospective study in which patients were recruited over a period of one year. Patients presenting with symptomatic pre-sternal keloids and requesting treatment but were unwilling to undergo surgical intervention were included in this study. Patients with a history of previous thoracic surgery were excluded. Baseline assessment and documentation of the lesion were performed. The study patients received three sessions of intralesional injections of a combination of triamcinolone acetonide and hyaluronidase at four weekly intervals. The final assessment was performed four weeks after the third session.

Results: The study included 47 lesions in 47 patients with ages of the patients ranging from 16 to 70 years. Pre-sternal keloids were found to be more common among males than females, with a male-to-female ratio of 2.35:1. Patients presented with pre-sternal keloids that had been present for varying periods ranging from three to 81 months. All of our 47 patients completed the three sessions of the treatment. Following the treatment, there was an improvement in the patient's symptoms, as evidenced by the reduction in the mean pruritis scores and pain scores. There was an overall reduction in the size of the lesion. The decrease in the height of the lesions was more evident than the reduction in the craniocaudal or transverse dimensions of the lesions. There were improvements in Vancouver Scar Scale (VSS) vascularity scores and pliability scores following the treatment.

Conclusion: We conclude that pre-sternal keloids should be considered as a distinct clinico-pathological entity. There are differences with regard to pathogenesis, clinical presentation, and management when compared to keloids elsewhere. Treatment with intralesional injections of a combination of triamcinolone acetonide and hyaluronidase effectively relieves the symptoms and may be considered in patients not willing to undergo surgical intervention. Recurrences can occur and need further treatments.

## Introduction

Keloid represents a pathological form of scarring. It is characterized by an overproduction of collagen and local fibroblast proliferation that occurs in response to dermal injury [1]. Patients suffering from keloids often complain of symptoms such as pruritus, pain, recurrent skin infections, and unesthetic appearance. The pathogenesis of keloids is influenced by various local, systemic, and genetic factors. The genetic susceptibility factors include single nucleotide polymorphisms [2]. Patients with multiple keloids tend to have a positive family history, and several genetic factors related to familial keloids have been reported [3].

Moreover, keloids are a rare occurrence in patients with Down's syndrome [4]. Keloids seem to exhibit a geographical variation in incidence; this may relate to population genetics and/or environmental factors: incidence ranges from 0.09% in the United Kingdom to as high as 16% in Congo [5]. Darker-skinned populations have a two- to 19-fold higher incidence of keloid than fair-skinned populations [6].

Although many keloids result from significant skin injuries such as those caused by trauma, burns, and surgery, they can also be induced by minor inflammations of the skin, such as acne and folliculitis. Keloids are a result of chronic inflammation in the reticular dermis. This is primarily driven by mechanical tension on the scar edges, and it can also be worsened by systemic factors, including hypertension, pregnancy, and conditions that are associated with high levels of circulating inflammatory cytokines [7,8]. Remarkably, the cyclical skin stretching that occurs during daily body movements is a substantial factor in the causation of keloids [9].

Keloids are particularly common in the anterior chest area; an earlier study showed that nearly 50% of all keloids occur in this location [10]. One of the reasons for this is that folliculitis and acne, known for triggering the development of keloids, are common on the anterior chest. The other reason is the tension load in this area due to the frequent movements of the upper limbs and the respiratory movements. These movements stretch the skin of the anterior chest horizontally. When this cyclical tension is imposed on the anterior chest wounds, there is an exacerbation and prolongation of the inflammation in the reticular dermis of the wound [9]. These stresses induce the growth of keloids along the prevailing lines of skin tension. This also explains why the pre-sternal keloids on the chest develop characteristic shapes [11]. For instance, some keloids are small and oval, while some are large and irregular.

Various treatments are used for pre-sternal keloids, such as intralesional injections of therapeutic agents like triamcinolone acetonide or 5-fluorouracil, silicone sheets, pressure therapy, laser treatment, and surgical excision with adjuvant radiotherapy [12-15]. In the pre-sternal region, the mobility of tissues is relatively limited; therefore, it is generally accepted that the reconstruction after resection of keloids in this region is challenging. Sometimes, after excising a pre-sternal keloid, the defect may need to be reconstructed with a local, regional, or distant flap. The clinician should choose the most appropriate treatment based on the individual patient's symptoms. These lesions have a high rate of recurrence after treatment due to multiple reasons [16-20].

The aim of our study was to understand the demographic factors, morphological features, and clinical presentation of pre-sternal keloids and evaluate the effectiveness of intralesional injections of a combination of triamcinolone acetonide and hyaluronidase in alleviating the symptoms associated with these lesions.

## Materials and methods

Study design

We performed a prospective study in which patients were recruited over a period of one year. The study was conducted at the Department of Burns and Plastic Surgery of a tertiary care institute in a non-metropolitan city in central India.

Inclusion criteria

Patients presenting with symptomatic pre-sternal keloids of any size but not willing to undergo surgical management were included in the study.

Exclusion criteria

Patients presenting with pre-sternal keloids caused by thoracic surgery were excluded from the study. Whenever a patient presented with an ulceration or active infection with pus discharge in a pre-sternal keloid, we treated the patient with local care and antibiotics as required and ensured that the ulcer healed or the infection subsided before initiating the intralesional injections.

Procedure

The Institutional Human Ethics Committee approved the study protocol. Informed consent was taken before administering the treatments. We recorded the patient demographic data, symptoms, and clinical features of the lesion at the baseline before administering the first dose of the treatment. Whenever multiple lesions were present, we considered the largest lesion as the index lesion in the respective patient and recorded its dimensions, features, and scores. We initially intended to use the Vancouver Scar Scale (VSS) to assess and grade the pre-sternal keloids [21]. During the study, we realized that some components of the VSS were not suitable for accurate application in pre-sternal keloids. Hence, we have considered specific components of the VSS in our study.

At each sitting, the study patients received intralesional injections of a combination of 1 mL of triamcinolone acetonide (40 mg/mL) and 1 mL of hyaluronidase (1500 IU/mL). After taking aseptic precautions, injections were given with a 30 G insulin syringe at multiple sites within the lesion until blanching was observed at each site. We did not use any local anesthesia during the procedure.

The second dose was given after four weeks, and the third dose was given after eight weeks of the first dose. Post-treatment assessment of the lesion and scoring were done 12 weeks after the first dose.

Primary study outcome and other variables

The primary outcome variables that were measured were the changes or otherwise in the symptoms and morphological features of the lesion as well as the treatment complications.

Statistical analysis

All the patient data were entered in a spreadsheet created in Microsoft Excel™ software (©Microsoft Corporation, Redmond, Washington, United States). Data analysis was done using Epi Info software version 7.2 (Epi Info™, Centers for Disease Control and Prevention, Atlanta, Georgia). Nominal variables were statistically described with frequencies and percentages. Continuous variables were described with means and standard deviations. Ordinal variables were also presented in the form of frequencies and percentages.

## Results

The demographic features of the patients are summarized in Table 1. The study included 47 lesions in 47 patients with ages ranging from 16 to 70 years. The mean age was 33.42 ± 13.21 (mean ± SD). There were 33 (70.21%) males and 14 (29.78%) females. Fitzpatrick skin type IV was the most common skin type in our patient group (n=19, 40.42%) [22]. A Setty pattern of pecto-sterno-infraclavicular (PSI) was the most frequent chest hair pattern seen (n=20, 42.55%) in our group of patients [23]. Table 2 describes the presenting features of the patients.

**Table 1:** Demographic characteristics of the study patients

S. No.	Characteristic	Frequency
1	Age (years)	Mean ± SD: 33.42 ± 13.21
		Range: 16-70
2	Sex	
	Male	33 (70.21%)
	Female	14 (29.78%)
	Male:female ratio	2.35:1
3	Fitzpatrick skin type	
	Type I	0
	Type II	0
	Type III	10 (21.27%)
	Type IV	19 (40.42%)
	Type V	16 (34.04%)
	Type VI	2 (0.04%)
4	Chest hair pattern (Setty patterns)	
	A: apilose	14 (29.78%)
	C: circumareolar	01 (0.02%)
	PSI: pecto-sterno-infraclavicular	20 (42.55%)
	CPS: circumareolo-pecto-sternal	03 (0.06%)
	P: pectoral	01 (0.02%)
	PI: pecto-infraclavicular	03 (0.06%)
	CS: circumareolo-sternal	03 (0.06%)
	S: sternal	01 (0.02%)
	PS: pecto-sternal	01 (0.02%)
5	Presence of comorbidities	
	No comorbidity	43 (91.48%)
	Hypertension	01 (0.02%)
	Hypertension/diabetes mellitus	01 (0.02%)
	Hypertension/coronary artery disease	01 (0.02%)
	Hypertension/diabetes mellitus/coronary artery disease	01 (0.02%)
6	History of previous treatment with triamcinolone acetonide	
	Present	17 (36.17%)
	Absent	30 (63.82%)

**Table 2:** Clinical characteristics of the study patients

S. No.	Characteristic	Frequency
1	Duration of pre-sternal keloid (months)	Mean ± SD: 20.57 ± 15.14
		Range: 3-81
2	Presenting symptoms	
	Pruritus	47 (100%)
	Pain	38 (80.85%)
	Esthetic concerns	37 (78.72%)
	Pus discharge	10 (21.27%)
	Ulcer over the keloid	06 (12.76%)
3	Recurrent symptoms	13 (27.65%)
4	Etiology	
	Spontaneous	22 (46.80%)
	Folliculitis	17 (36.17%)
	Post-surgery	06 (12.76%)
	Post-trauma	02 (0.04%)

Patients presented with pre-sternal keloids that had been present for varying periods ranging from 3 to 81 months (mean ± SD: 20.57 ± 15.14). All the patients had pruritis as one of their presenting complaints, pain of varying degrees was present in 38 (80.85%) patients. Thirty-seven (78.72%) patients complained about the unesthetic appearance. Ten (21.27%) patients presented with pus discharge, and six (12.76%) patients presented with an ulcer over the keloid. Thirteen (27.65%) patients suffered from recurrent symptoms. In 22 (46.80%) patients, pre-sternal keloids seemed to occur spontaneously without any cause, while folliculitis seemed to be the cause in 17 (36.17%) patients. Table 3 summarizes the morphological features of the pre-sternal keloids in our patients.

**Table 3:** Morphological characteristics of the pre-sternal keloids

S. No.	Characteristic	Frequency
1	Number of lesions in a given patient	
	01	38 (80.85%)
	02	05 (10.63%)
	03	03 (0.06%)
	04	01 (0.02%)
2	Location of the pre-sternal keloid	
	Manubrium	07 (14.89%)
	Manubrium and body upper-third	01 (0.02%)
	Body upper-third	15 (31.91%)
	Body upper-third and middle-third	01 (0.02%)
	Body middle-third	13 (27.65%)
	Body middle-third and lower-third	02 (0.04%)
	Body lower-third	06 (12.76%)
	Body lower-third and xiphisternum	01 (0.02%)
	Xiphisternum and epigastrium	01 (0.02%)
3	Shape	
	Oval	15 (31.91%)
	Rectangular	06 (12.76%)
	Trapezoid	07 (14.89%)
	Dumbell	07 (14.89%)
	Irregular	05 (10.63%)
	Butterfly	07 (14.89%)
4	Presence of visible sinuses in the pre-sternal keloid	
	Present	17 (36.17%)
	Absent	30 (63.82%)

**Figure 1:**
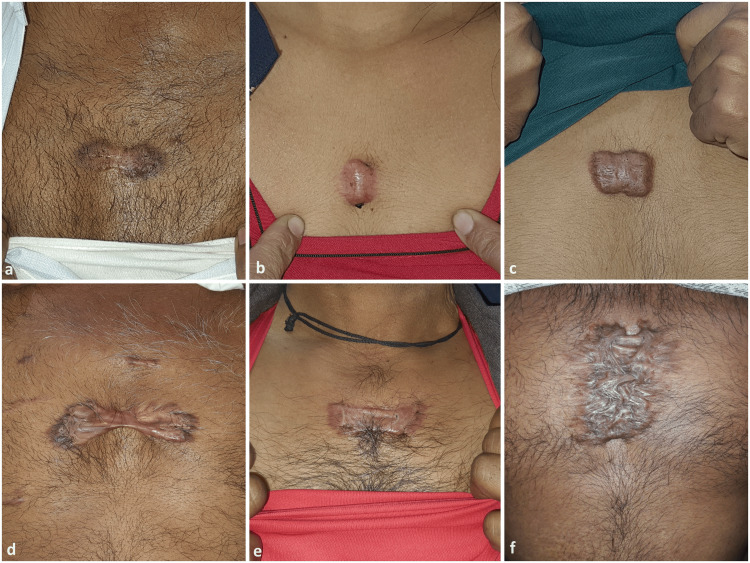
The spectrum of shapes of pre-sternal keloids in our study patients a: dumbell; b: oval; c: rectangular; d: butterfly; e: trapezoid; f: irregular

There were a total of 61 lesions in 47 patients. The majority of the patients (n=38, 80.85%) presented with a single pre-sternal keloid. Considering the location of the lesion, the highest number of the lesions were located in the upper third of the body of the sternum (n=15, 31.91%), closely followed by the middle third (n=13, 27.65%). Pre-sternal keloids presented in varying shapes like oval, rectangular, trapezoid, butterfly-shaped, and dumbbell-shaped lesions; some were irregularly shaped. Figure 1 shows the varied spectrum of keloid shapes that were seen in our patients.

Seventeen (36.17%) patients had visible sinuses in the lesion at presentation, most of them with growing hair. Seventeen (36.17%) patients had a history of receiving intralesional injections of triamcinolone acetonide before. Forty-three (91.48%) patients in our study had no comorbidities. The mean pre-treatment and post-treatment values of the different variables measured are given in Table 4.

**Table 4:** Mean pre-treatment and post-treatment scores of the measured variables VAS: Visual Analog Scale; VSS: Vancouver Scar Scale

Study variable	Baseline (mean ± SD)	Final assessment (four weeks after the third treatment session)
Pruritis score	2.74 ± 0.82	0.78 ± 0.74
Pain (VAS score)	4.97 ± 1.42	1.55 ± 0.95
Maximum height of the lesion (mm)	5.76 ± 2.23	2.25 ± 1.40
Maximum vertical dimension (mm)	31.29 ± 18.31	27.25 ± 17.04
Maximum transverse dimension (mm)	49.38 ± 28.13	42.59 ± 26.68
VSS height score	2.53 ± 0.49	1.53 ± 0.49
VSS vascularity score	2.65 ± 0.51	0.97 ± 0.60
VSS pliability score	2.65 ± 0.47	1.27 ± 0.67

**Figure 2:**
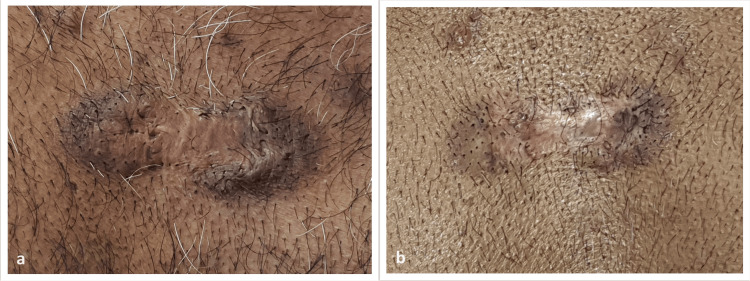
Pre-treatment and post-treatment picture in a pre-sternal keloid a: pre-treatment picture; b: post-treatment picture

**Figure 3:**
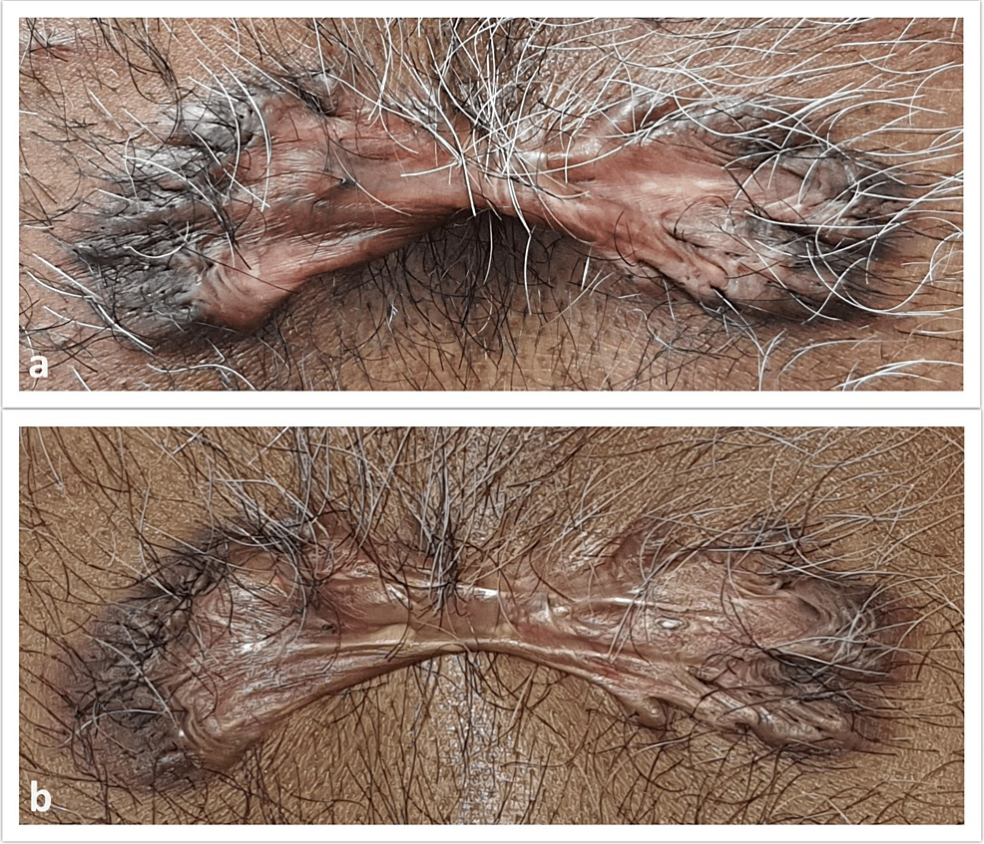
Pre-treatment and post-treatment picture in another pre-sternal keloid a: pre-treatment picture; b: post-treatment picture

All of our 47 patients completed three sessions of the treatment. Figure 2 and Figure 3 show the pre-treatment and post-treatment pictures of two patients in our study.

Table 5 summarizes the complications of the treatment and their frequency. There were no complications in 21 (44.68%) patients. The most common complication reported was an unequal response across the lesion to the treatment (n=17, 36.17%).

**Table 5:** Complications

Complication	Frequency
No complications	21 (44.68%)
Unequal response across the lesion	17 (36.17%)
Hypopigmentation	09 (19.14%)
Telangiectasia	07 (14.89%)

## Discussion

The terms "pre-sternal keloids" and "chest keloids" have been used interchangeably in the literature. According to our observations, keloids in the pre-sternal region behave differently from those in the lateral chest region. Therefore, we have differentiated the broad entity of chest keloids into pre-sternal keloids and lateral chest keloids. This differentiation is based on whether the majority of the lesion (>50 %) lies in the pre-sternal region or otherwise. Moreover, the tensile forces acting on a centrally situated lesion in the chest differ from those acting on a keloid situated more laterally. Our study focuses mainly on pre-sternal keloids.

The cyclical skin stretching during bodily movements is a strong factor for keloidogenesis [9,24]. The pre-sternal skin is especially prone to such mechanical stresses, given its constant motion due to arm movements. These mechanical stresses cause keloids to grow along the lines of predominant skin tension; this explains the reason for pre-sternal keloids adopting characteristic shapes [11]. We have attempted to group these lesions based on their location on the respective part of the sternum and on their shape. The different shapes of pre-sternal keloids may result from the tensile forces acting on the lesion, which may be, in part, related to their location on the sternum.

In our study, pre-sternal keloids were found to be more common among males than females, with a male-to-female ratio of 2.35:1. One of the possible explanations for this is the presence of thick chest hair in males, which makes them more prone to folliculitis, which can lead to keloidogenesis. We have also found out that the PSI chest hair pattern was mostly present in males presenting with pre-sternal keloids in our study. According to the existing literature, there is a two- to 19-fold higher incidence of keloids in dark-skinned populations than in lighter-skinned populations [6]. Our findings are consistent with this; 78.72% (n=37) of patients had a Fitzpatrick IV, V, or VI skin type.

Although many keloids arise from skin injuries induced by trauma, burns, or surgery, they can also be triggered by minor inflammations of the skin such as acne and folliculitis. Half of these keloids occur on the anterior chest wall area [10]. Our study found that a history of folliculitis was present in 36.17% (n=17) patients, while no apparent cause could be found in 46.80% (n=22) patients.

According to the literature, patients with pre-sternal keloids present with symptoms like itching, pain, skin infections, and also cosmetic concerns, as these are located in the exposed regions of the chest [25]. We also report identical findings in our study; 78.72% (n=37) of our patients had esthetic concerns due to the pre-sternal keloid.

21.27% (n=10) of patients presented with pus discharge and 12.76% (n=6) of patients presented with an ulcer over the keloid in our study. These patients were initially managed conservatively, and infection control/ulcer healing was ensured before initiating the intralesional treatments. No patient developed any ulceration during the course of the treatment.

We have identified that in most of the pre-sternal keloids, particularly the large-sized ones, the inflammatory activity was more in the periphery of the lesion and the central part of the the lesion was relatively quiescent. Thirteen (27.65%) patients in our study suffered from recurrent symptoms at presentation. There is a multitude of causes that contribute to the higher rate of recurrence in pre-sternal keloids [16-20].

We have found that following three sessions of intralesional triamcinolone acetonide and hyaluronidase injections, there was an improvement in the patients' symptoms, as evidenced by the reduction in the mean pruritis scores and pain scores. There was an overall reduction in the size of the lesion. The decrease in the height of the lesions was more evident than the reduction in the craniocaudal or transverse dimensions of the lesions. There were reductions in VSS vascularity scores and pliability scores following the treatment, indicating decreased inflammatory activity within the pre-sternal keloid. This suggests that the intralesional treatment with a combination of triamcinolone acetonide and hyaluronidase was effective in reducing the symptoms of pre-sternal keloids like pruritis and pain, but it cannot lead to a significant reduction in the size of large-sized or long-standing lesions.

Intralesional injections of triamcinolone acetonide and hyaluronidase were not without complications. Although hypopigmentation was seen in 19.14% (n=9) patients and telangiectasias were seen in 14.89% (n=7), the most common complication seen in our patients was an unequal response across the extent of lesion that was seen in 36.17% (n=17) patients. This, to some extent, can be explained by the large-sized lesions that were included in the study.

The last follow-up was six months after the third session of treatment. At the last follow-up visit, 72.34% (n=34) patients had no significant recurrence of symptoms, while 27.65% (n=13) patients had a recurrence of one or more symptoms, needing treatment.

Although conservative therapy has been recommended as the first-line treatment for keloids, surgical excision should be considered, especially in those with a poor response to conservative treatments. One of the critical elements of surgery is to reduce the wound tension, which has been considered a dominant factor in keloidogenesis. A small-sized pre-sternal keloid can be excised and the wound closed primarily. When it is difficult to close the wound primarily, local flaps are the best option. Freestyle or pedicled propeller flaps based on the IMA (internal mammary artery) perforators and keystone perforator island flaps are the options that can be considered. Tissue expansion can also be considered for large lesions [26]. Free flaps have also been used successfully for the reconstruction of the defects after the excision of pre-sternal keloids [27]. After any form of surgical excision, adjuvant treatments should be used to prevent recurrences.

In our study, we have seen that certain chest hair patterns were more frequent in patients with pre-sternal keloids. Thick and dense chest hair may have a role in the causation of pre-sternal keloids. It may also aggravate the symptoms, cause difficulty in maintaining proper hygiene, and can cause repeated infections in pre-sternal keloids. Thus, there may be some benefit in recommending laser hair reduction in these patients.

The limitations of our study include a small sample size and a relatively short period of follow-up (six months). Further studies with a larger set of patients are required to address the issue of a smaller sample size. Since pre-sternal keloids are known for their propensity to recur, a longer follow-up period of one year or more may provide more useful information about the behavior of these lesions.

## Conclusions

We conclude that pre-sternal keloids should be considered as a distinct clinico-pathological entity. There are differences with regard to pathogenesis, clinical presentation, and management when compared to keloids elsewhere. Multiple treatment options are available, and treatment should be individualized for each patient according to the patient's features. Intralesional injections of a combination of triamcinolone acetonide and hyaluronidase result in effective relief from the symptoms and may be considered in patients not willing to undergo surgical intervention. Recurrences can occur and need further treatments.
